# Characterization of the flavor profile of Hulatang using GC-IMS coupled with sensory analysis

**DOI:** 10.3389/fnut.2024.1461224

**Published:** 2024-08-29

**Authors:** Jing Yan, Heng Wang, Bing Yang, Wanli Zhang, Zhenxia Cao, Penghui Zhao, Zijie Dong, Fazheng Ren, Lishui Chen

**Affiliations:** Food Laboratory of Zhong Yuan, Luohe, China

**Keywords:** Hulatang, HS-GC-IMS, volatile components, sensory evaluation, correlation, aroma components

## Abstract

**Background:**

Hulatang is a traditional specialty snack in Henan, China, and is well known for its unique flavor.

**Methods:**

In this study, the volatile organic compounds (VOCs) in four kinds of Hulatang from two representative regions in Henan Province (Xiaoyaozhen and Beiwudu) were evaluated using headspace-gas chromatography-ion mobility spectrometry (HS-GC-IMS).

**Results:**

The results showed that Xiaoyaozhen Hulatang exhibited more ethers, fewer terpenes and ketones than Beiwudu Hulatang. Additionally, Hulatang from different regions were classified using the orthogonal partial least squares-discriminant analysis (OPLS-DA) based on GC-IMS data. Twenty aroma substances were selected as the potential markers using the variable importance in the projection (VIP) variable selection method. Additionally, fifteen aroma components significantly contributing to the aroma of Hulatang were screened using the relative odor activity value (ROAV) (ROAV > 1). Combined with the sensory score results, twelve key substances with significant correlation with odor perception were selected. The flavor characteristics of the key substances revealed that the flavor of Hulatang was mainly composed of volatile components with camphor, green, almond, fatty, spicy, herbal, vegetable, fruity, floral, musty, and solvent aromas.

**Conclusion:**

Overall, the experimental results provide a theoretical basis for evaluating the flavor characteristics of Hulatang from different regions using GC-IMS.

## 1 Introduction

For hundreds of years, Hulatang has been one of the most renowned Chinese foods in Henan province, especially in the Song and Qing Dynasties (1). This recipe is renowned for its meticulous preparation, integrating over 20 types of medicinal and culinary ingredients, such as white pepper, black pepper, star anise, Sichuan pepper, etc. (2). The ingredients are exquisite, and the soup is fresh and flavorful, with a balance of spiciness and a lingering taste. According to the geographical location, Hulatang can be divided into two main regions: Beiwudu Hulatang and Xiaoyaozhen Hulatang, which are located upstream and downstream of the Shahe River. Beiwudu Hulatang is recognized for its abundance of meat and mild taste, while Xiaoyaozhen Hulatang is recognized for its diverse ingredients and robust spicy flavor (3). However, the aroma of different regions of Hulatang exhibits certain differences due to the differences in recipes and production processes.

In recent years, although there has been significant growth in the Hulatang industry (2), research on Hulatang is scarce, restricting the standardization and industrial production of Hulatang products. Flavor contributes to sensory characteristics, and aroma and taste are important factors influencing consumer preferences (4). So far, several multivariate statistical techniques, such as principal component analysis (PCA), orthogonal partial least squares-discriminant analysis (OPLS-DA), and cluster analysis, have been developed to characterize the volatile compound patterns corresponding to specific sensory aroma profiles (5).

In this regard, gas chromatography-ion mobility spectrometry (GC-IMS) has emerged as a novel separation and detection technique (6–8). Gas chromatography coupled with ion mobility spectrometry (GC-IMS) can meet the analytical needs of high resolution and low detection limits, making it a potential approach for flavor substance detection (9). For instance, GC-IMS analysis technology has been widely used for volatile flavor compound detection, quality detection and analysis, and product classification of various foods (10–12).

Based on the unique flavor of Hulatang, the present study selected the two representative regions of Hulatang, Xiaoyaozhen and Beiwudu, as the research objects, and the aroma profiles of Hulatang were characterized using GC-IMS. Additionally, a fingerprint of the volatile compounds was established, and the differences in aroma components in Hulatang were explored using PCA and OPLS-DA analysis. The key aroma components were screened using the relative odor activity values (ROAVs) in combination with the sensory evaluation results. Furthermore, the difference in sensory quality characteristics and the composition of flavor substances in Hulatang were investigated to broaden the ideas for the rapid and efficient analysis of volatile compounds in Hulatang.

## 2 Materials and methods

### 2.1 Material

Based on the market research, two representative products, Beiwudu Hulatang and Xiaoyaozhen Hulatang, were selected, and their basic information is shown in Supplementary Table S1. Refer to the edible method of the product to cook, add water to the pot, after the water boils, add various packets, add starch for thickening and boil for 2–3 min, take the soup and set aside. Each sample was stored at room temperature and cooked for use before instrument or sensory analysis.

### 2.2 Headspace gas chromatography-ion mobility spectrometry (HS-GC-IMS) analysis

The volatile organic compounds (VOCs) in Hulatang samples were analyzed using the GC-IMS method composed of Agilent 490 gas chromatography (Agilent Technologies, Palo Alto, CA, USA) and IMS instrument (FlavourSpec®, Gesellschaft für Analytische Sensorsysteme mbH, Dortmund, Germany) and equipped with a PAL3 Automatic sampler (CTC Analytics AG Company, Switzerland). Before GC-IMS analysis, each freshly cooked Hulatang sample (2 g) was transferred into a 20-mL headspace vial and incubated at an oscillating heating mode (60°C) with a speed of 500 rpm for 15 min. Then, the headspace was injected by the PAL3 sampler automatically with an injection volume of 500 μL and injector temperature of 85°C. The injection method was performed according to the previously reported method with slight modification (13).

For GC detection, the VOCs were separated by an MXT-WAX capillary column (15 m × 0.53 mm, 1.0 μm) with a column temperature fixed at 60°C. High-purity nitrogen (≥ 99.999%) was used as the carrier gas with an initial flow rate of 2.0 mL/min for 2 min, which increased to 10 mL/min within 8 min, and then increased to 100 mL/min within 10 min and maintained at 150 mL/min for 10 min. Nitrogen (≥ 99.999% purity) was used as the drift gas with a flow rate of 150 mL/min, the volatiles were ionized in the IMS ionization chamber (positive ion mode) and the ions were driven to a 9.8 cm migration tube with a nitrogen flow at 45°C (10). The retention index (RI) of each volatile compound was calculated by the Laboratory Analytical Viewer (LAV) using n-ketones C4-C9 (Sinopharm Chemical Reagent Beijing Co., ltd., Beijing, China) as external references. The volatile compounds were identified based on the retention index (RI) and drift time (RIP relative) of the standards in the GC-IMS Library. The Reporter plug-in and Gallery Plot plug-in were used to form the spectrogram and volatile fingerprints of Hulatang samples.

### 2.3 Sensory evaluation

Thirty students (aged 20–30 years) majoring in food with no rhinitis and no smoking were selected for sensory evaluation training of Hulatang, and triangulation test was used for the screening of sensory evaluators. Each evaluator was presented with 3 coded samples of Hulatang, two of which were identical, and the evaluators were asked to taste each sample from left to right and select a different one (14). A sensory evaluation team of 20 trained individuals (10 females and 10 males) was screened. All sensory tests were done in the sensory assessment room. Before the start of the formal experiment, the panelists were informed of the objectives of the participation assessment, detailed experimental procedures, and sensory requirements. Four freshly cooked Hulatang samples were analyzed from the five dimensions of flavor, taste, color, mouthfeel, and likeability, and each sample was randomly coded with a different three-digit random code and presented to the panelists in a colorless transparent bowl. Mouthwash with tasteless and odorless water when tasting different samples. The specific scoring criteria are shown in Supplementary Table S2.

### 2.4 Calculating ROAV

The key flavor substances in Hulatang were determined using the ROAV value following the method of Xi et al. (15). The relative odor activity value (ROAV) indicates the contribution of the corresponding volatile flavor compounds to the overall aroma of the sample (16). The ROAV values were calculated as follows:


$$\mathrm{ROAV}\approx 100\times \left(\frac{\mathrm{Ci}}{\mathrm{Cmax}}\right)\times \left(\frac{\mathrm{Tmax}}{Ti}\right)$$

where Ci and Ti represent the relative percentage content of the target volatile compound and the corresponding odor threshold in water, respectively, and Cmax and Tmax represent the relative percentage content of the compound with the highest odor activity value and the corresponding odor threshold in water, respectively.

### 2.5 Statistical analysis

The HS-GC-IMS data was processed by the Laboratory Analytical Viewer (LAV, G.A.S., Dortmund, Germany) using three plug-ins and GC × IMS Library Search (NIST database and IMS database). The topographic plots and fingerprints of volatile compounds were established by plugins of Reporter and Gallery Plot (G.A.S., Dortmund, Germany). The significance analysis was performed using the SAS System for Windows V8 software, and the data were expressed as mean ± standard deviation. *p* < 0.05 was considered statistically significant. The relative content of each volatile compound was calculated using the peak area normalization method. Multiple statistical analysis was performed using SIMCA 14.1 for PCA, OPLS-DA, and the significance of the projection of the variables. Pearson correlation analysis was performed using IBM SPSS Statistic 26.0 software. Figures were generated using GraphPad Prism 8.0. All measurements were performed in triplicate.

## 3 Results and discussion

### 3.1 Analysis of volatile components in Hulatang

**Figure 1:**
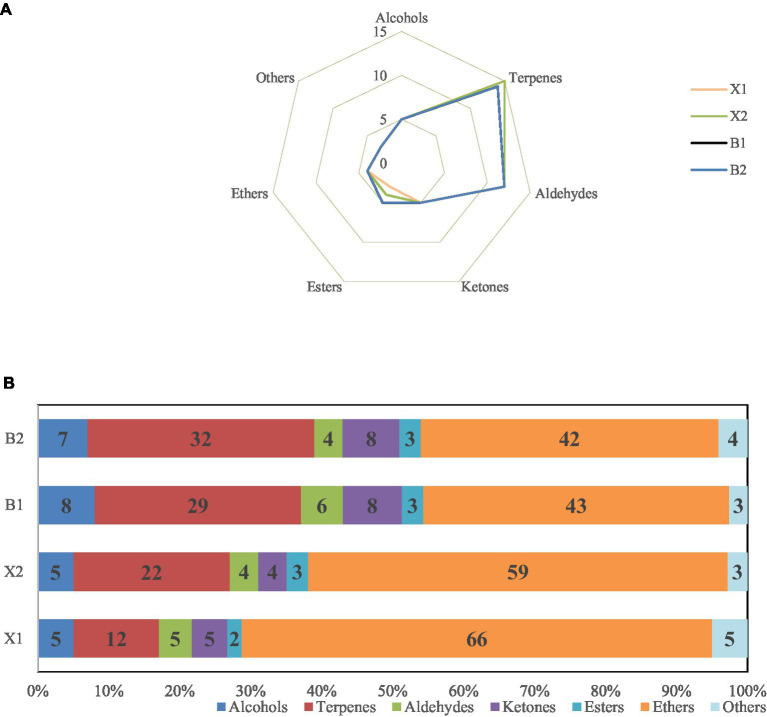
**(A)** Types and quantities of volatile components in different Hulatang samples. **(B)** Relative contents of volatile components in different Hulatang samples.

A total of 75 signal peaks were detected in the four varieties of Hulatang samples, of which 49 volatile components were identified (monomers and dimers were only calculated once), including 5 alcohols, 15 terpenes, 12 aldehydes, 5 ketones, 5 esters, 4 ethers, and 3 other compounds (Supplementary Table S3; Figure 1A). The results showed that terpenes and aldehydes accounted for most of the volatiles in Hulatang. Terpenes are mainly found in spices, such as pepper and Huajiao (17, 18), which are also the main ingredients of Hulatang (2). Most straight-chain aldehydes were derived from the oxidation of unsaturated fatty acid in the samples, such as hexanal, octenal, pentanal or heptanal etc. (19), which might be due to the heating of the oil and meat in the raw materials of Hulatang.

As shown in Figure 1B, the Hulatang varieties exhibited distinct volatile compounds, but the samples of Hulatang of the same genre showed the same trends. Only 4 ethers were detected in Hulatang, but the content was the highest, followed by terpenes. The content of ethers in X1 and X2 samples was significantly higher than in B1 and B2, and the corresponding content of terpenes was significantly lower, which might be associated with the flavor characteristics of Hulatang between different regions. The majority of ethers are derived from spices, and it is an easy volatile and release compound (20). Terpenes are important components of volatile components and play an important role in flavor formation due to their low thresholds (21). The differences between these volatile components might be attributed to the differences in aroma between regions. Similar results were reported in truffles and tea (22, 23).

### 3.2 GC-IMS profile analysis of volatile components in Hulatang

**Figure 2:**
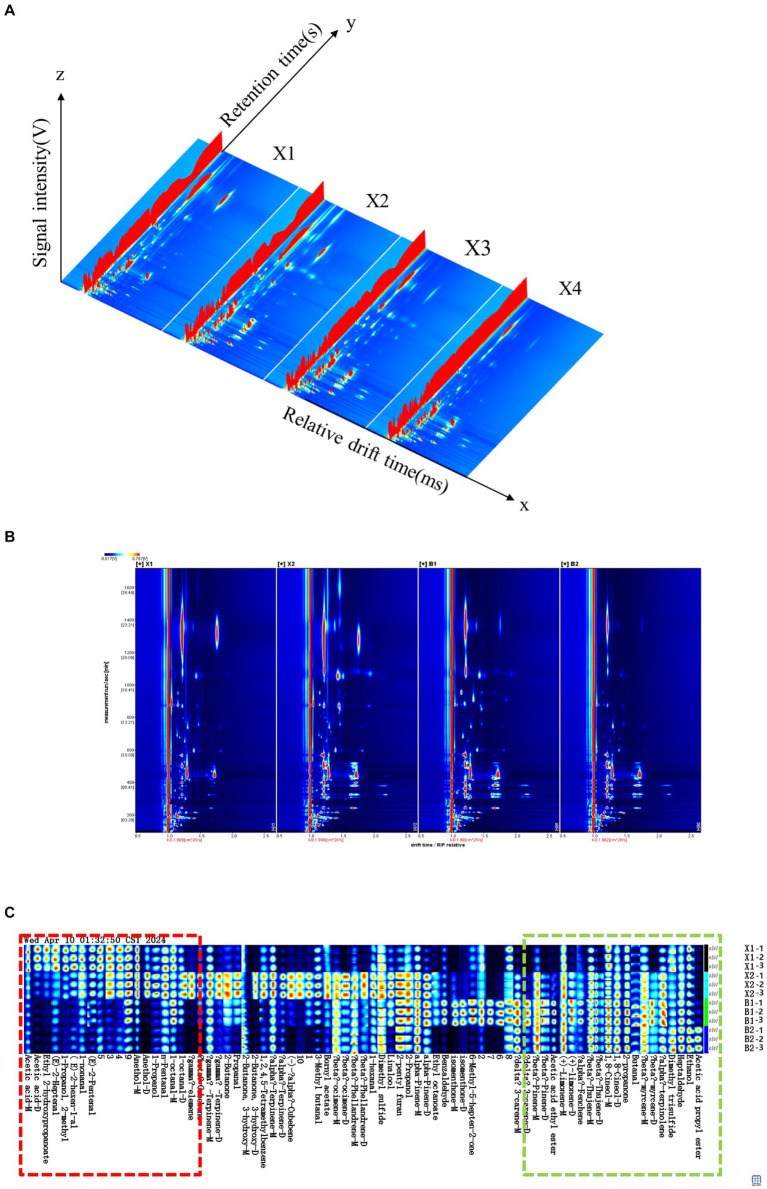
GC-IMS analysis of four different Hulatang samples. **(A)** 3D-topographic plots; **(B)** 2D-topographic plots; **(C)** fingerprints of volatile compounds.

The GC-IMS profiles of four Hulatang samples are illustrated in Figure 2. As shown in Figure 2A, the GC-IMS analysis of Hulatang volatiles resulted in a 3D-topographic plot using the Reporter plug-in, with three axes representing the ion migration time (*X* axis), the retention time (*Y* axis), and the ion peak intensity for quantification (*Z* axis), respectively (24). The 2D-topographic spectra of volatile compounds in Hulatang samples are shown in Figure 2B. As shown in Figures 2A,B, the GC-IMS spectra background is blue, and the red vertical line at the horizontal coordinate 1.0 is the reactive ion peak (RIP). Each point on both sides of the RIP peak represents a volatile compound. From blue to red, the darker color indicates the greater peak intensity and the higher concentration of the corresponding volatile compound (25).

All volatile compounds identified in GC-IMS spectra were selected to generate a volatile fingerprint to observe the differences in volatile compound profile among four Hulatang samples, as shown in Figure 2C. As shown in Figure 2C, each row represents all the signal peaks in a sample, and each column reveals the signal intensity of the same volatile organic compounds in different samples. The Hulatang sample X2 had a higher content of volatile components, including (−)-α-cubebene, γ-elemene, γ-terpinolene, α-terpinene, and 3-hydroxy-2-butanone. The X1 sample had higher contents of (E)-2-heptenal, ethyl 2-hydroxypropanoate, and acetic acid. The content of 3-carene, 6-methyl-5-hepten-2-one, and isomenthone in B1 were significantly higher than those in other samples. B2 contained the highest amount of acetic acid propyl ester.

Further comparison indicated that the distribution of volatile substances in different regions of Hulatang was inconsistent with some common areas and distinct characteristic peaks. The red rectangle indicates the characteristics of flavor substances in Xiaoyaozhen Hulatang (X1 and X2), and the contents of flavor substances were higher than in Beiwudu Hulatang (B1 and B2), mainly including acetic acid, 2- hydroxypropanoate, (E)-2-heptenal, 2-methyl-1-propanol, (E)-2-hexenal, 1-nonanal, (E)-2-pentenal, anethol, 1-propanol, n-pentanal, 1-octanal, and γ-Elemene. Additionally, 14 identified compounds were observed as the dominant volatiles (3-carene, β-pinene, acetic acid ethyl ester, (+)-limonene, α-fenchene, β-thujene, 1,8-cineol, 2-propanone, butanal, α-terpinolene, dimethyl trisulfide, heptaldehyde, ethanol, and acetic acid propyl ester) in Beiwudu Hulatang (B1 and B2) samples, as labeled with green rectangle in Figure 2C. The obtained information suggested that different regions of Hulatang can be distinguished according to the distribution characteristics of volatiles characterized by GC-IMS. It is reported that GC-IMS has been successfully used in the discrimination of various food products through volatile compound analysis (26).

### 3.3 Similarity analysis of volatile organic compounds in different regions of Hulatang

**Figure 3:**
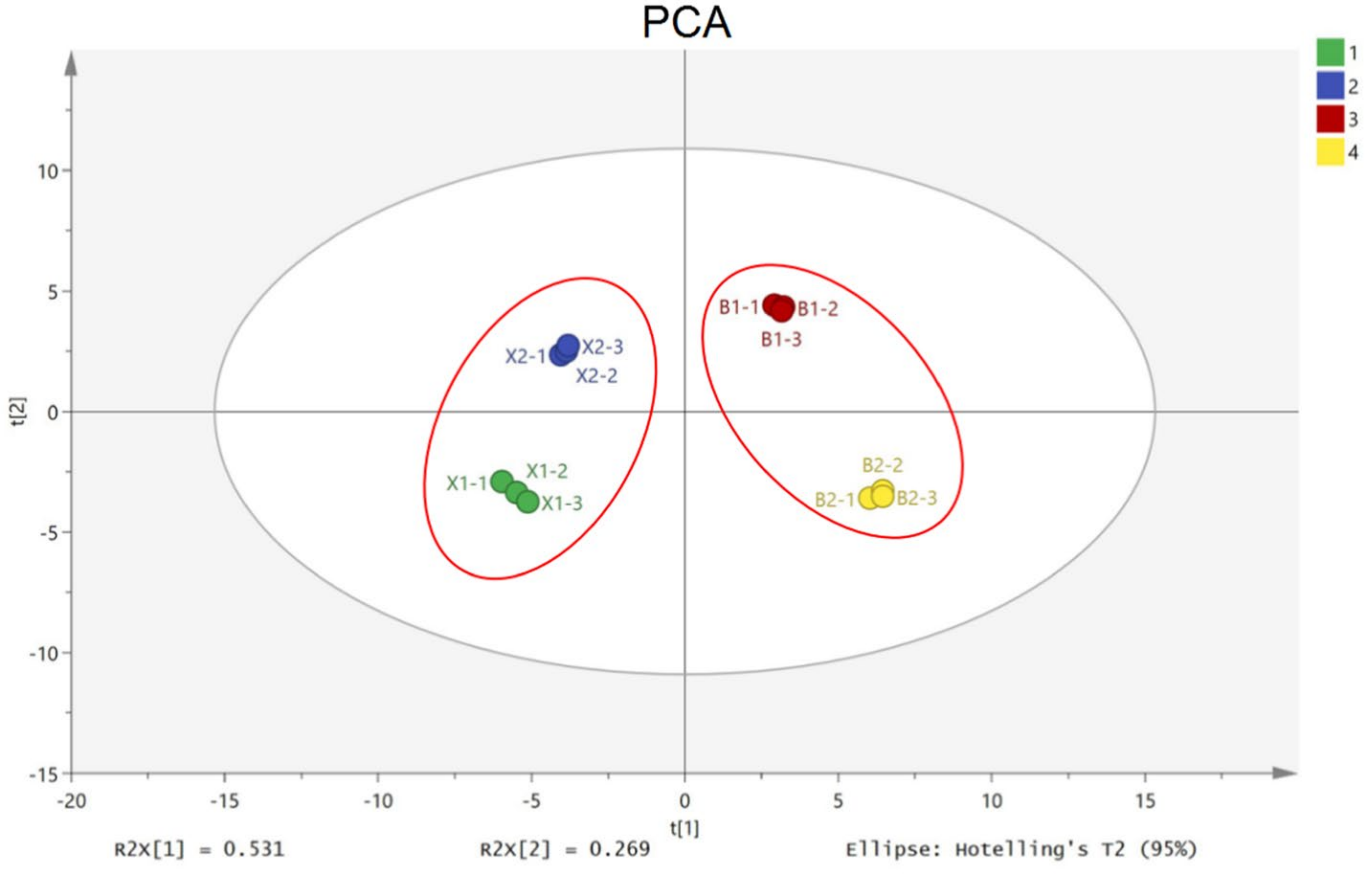
PCA scatter plot of the different Hulatang samples.

The regularity and differences among aroma profiles of Hulatang samples were evaluated using the principal component analysis (PCA) (27). The total contribution ratio of the first two principal components reached 80% (PC1 and PC2 showed contribution rates of 53 and 27%, respectively) and was higher than the total ratio of 60% (Figure 3), which was sufficient to characterize the similarities between different samples (28). Based on the PCA distribution map, the Hulatang samples from the same genre were close to each other (Figure 3). As shown in Figure 3, green, blue, red, and yellow represent the X1, X2, B1, and B2 samples, respectively. However, the Hulatang samples of different factions were distributed separately (Figure 3). The X1 and X2 samples were clustered in the left area and B1and B2 were clustered in the right area (Figure 3). Consequently, GC-IMS combined with PCA presented good efficiency for classifying Hulatang from different regions in China. The differences in volatile profiles among Hulatang might be attributed to their types of raw materials, sources, processing techniques, etc.

### 3.4 Evaluation and analysis of the OPLS-DA model

**Figure 4:**
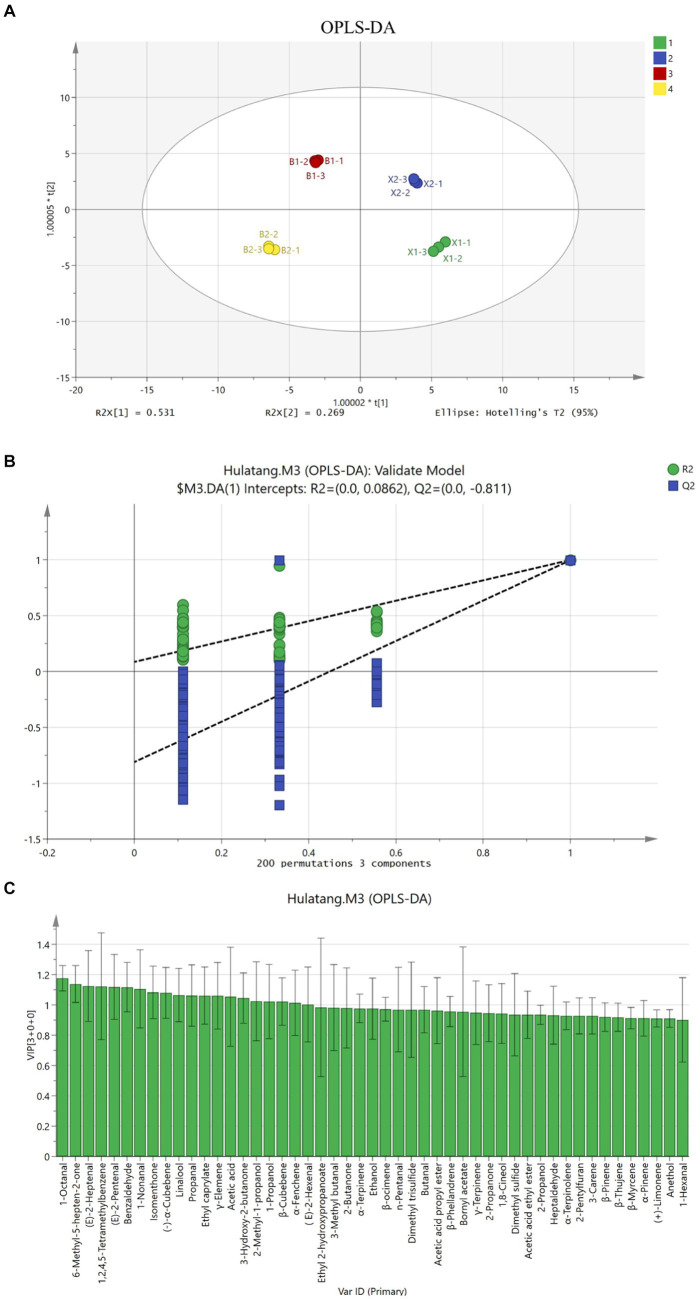
OPLS-DA analysis of different Hulatang samples based on GC-IMS. **(A)** OPLS-DA score plot. **(B)** Model cross-validation results. **(C)** VIP scores of OPLS-DA.

The OPLS-DA model is a supervised statistical method for discriminant analysis, which can obtain the classification information based on one principal component, simplify the model, and realize the prediction of the sample class (29). Based on the qualitative and quantitative results, OPLS-DA was performed to identify the differences in volatile compounds in Hulatang samples. Q2 (predictive power) was used to evaluate the predictive capacity of the model, and R2X and R2Y (fitting ability) were used to evaluate the goodness of fit and reliability. These parameters can range from 0 to 1. The closer the parameter is to 1, the more predictable or interpretable the model is (30). In this study, R2X = 0.978, R2Y = 0.997, Q2 = 0.993, and the model fitted well with acceptable predictability. As shown in Figure 4A, the samples were separated, the distribution of X1 and X2 samples was very close, and the B1 and B2 samples were located in the same region. The classification results were consistent with the PCA scatter plot. In summary, the samples of Xiaoyaozhen and Beiwudu Hulatang were well distinguished. The displacement test was used for further validation of the model, and only the successful model in the displacement test was used for data visualization and VIP analysis (31). After 200 displacement tests, twenty variables were found to contribute significantly (VIP >1), including three aldehydes (linalool, 1-propanol and 2-methyl-1-propanol), four terpenes (β-cubebene, (−)-α-cubebene, γ-elemene and α-fenchene), seven aldehydes (benzaldehyde, 1-nonanal, (E)-2-heptenal, 1-octanal, (E)-2-pentenal, (E)-2-hexenal and propanal), three ketones (6-methyl-5-hepten-2-one, isomenthone and 3-hydroxy-2-butanone), one esters (ethyl caprylate), one acid (acetic acid), and one aromatic 1,2,4,5-tetramethylbenzene.

### 3.5 Analysis of the key aroma compounds in Hulatang samples using ROAVs

The odor threshold refers to the lowest concentration of a particular volatile organic compound that a person can perceive. If the concentration of the volatile organic compounds is constant, the lower the aroma threshold, the greater the aroma contribution. ROAV has been widely used to quantify the aroma contribution of compounds (32, 33). Generally, if the ROAV of a compound is not less than 1, it contributes to the overall aroma. The greater the ROAVs, the greater the individual contribution of the compound. ROAVs can accurately reflect the effect of the aroma component on the overall aroma, despite the possibility of aroma synergy or suppression under the co-existence of some species (34). Table 1 lists all the compounds with their thresholds. A total of 15 key volatile organic compounds with ROAV >1 were screened. Notably, 1,8-Cineol (ROAV = 100 in the X1, X2, B1 and B2), also known as eucalyptol, was the most prominent contributor to the aroma of Hulatang samples. 1,8-Cineol has spice-like aromatics (35), endowing Hulatang with camphor, cool, and mint aroma (36). Among substances with a high ROAV value and a significant contribution to the aroma of Hulatang samples, dimethyl trisulfide and dimethyl sulfide mainly provided flavors similar to vegetables (37, 38), while anethol and β-myrcene mainly provided herbal flavors (39, 40). 1-octanal and 1-nonanal provided green and citrus notes (37, 38), linalool provided floral and citrus notes (41), and 3-methyl butanal provided malty and almond notes (42).

**Table 1:** The relative contents and relative odor activity values of aroma substances in Hulatang.

			Relative amount/%				ROAV			
Compound	Aroma attributes	Odor threshold (μg/kg)	X1	X2	B1	B2	X1	X2	B1	B2
Linalool	Floral, citrus	4.4	2.03 ± 0.05^c^	3.82 ± 0.17^b^	5.24 ± 0.06^a^	1.93 ± 0.09^c^	6.53	18.18	12.81	3.47
1-Propanol	Alcoholic	8505.6	0.14 ± 0.00^a^	0.12 ± 0.00^a^	0.08 ± 0.01^b^	0.12 ± 0.00^a^	<0.01	<0.01	<0.01	<0.01
2-Methyl-1-propanol	Bitter	7,000	0.24 ± 0.01^a^	0.08 ± 0.01^b^	0.06 ± 0.01^b^	0.10 ± 0.01^b^	<0.01	<0.01	<0.01	<0.01
Ethanol	Alcoholic	950,000	2.76 ± 0.17^b^	1.50 ± 0.05^c^	2.54 ± 0.04^b^	5.03 ± 0.08^a^	<0.01	<0.01	<0.01	<0.01
2-Propanol	Alcoholic, musty, woody	9787.9	0.12 ± 0.00^b^	0.12 ± 0.00^b^	0.24 ± 0.01^a^	0.22 ± 0.01^a^	<0.01	<0.01	<0.01	<0.01
β-Cubebene	Citrus, fruit, flower	NA	1.43 ± 0.07^c^	2.79 ± 0.02^a^	2.05 ± 0.02^b^	0.65 ± 0.13^d^	n.c.	n.c.	n.c.	n.c.
(−)-α-Cubebene	Herb, wax	NA	0.15 ± 0.00^c^	0.60 ± 0.02^a^	0.28 ± 0.01^b^	0.11 ± 0.03^c^	n.c.	n.c.	n.c.	n.c.
γ-Elemene	Spicy, fennel	NA	0.68 ± 0.01^b^	2.41 ± 0.07^a^	–	–	n.c.	n.c.	n.c.	n.c.
α-Terpinolene	Terpenic, green, woody	200	1.07 ± 0.05^b^	1.31 ± 0.03^b^	2.54 ± 0.03^a^	2.42 ± 0.05^a^	0.08	0.14	0.14	0.10
α-Fenchene	Camphoraceous, sweet	240	0.04 ± 0.01^c^	0.03 ± 0.01^c^	0.09 ± 0.01^a^	0.07 ± 0.00^b^	<0.01	<0.01	<0.01	<0.01
β-ocimene	Herb, floral	34	0.30 ± 0.02^c^	1.20 ± 0.02^b^	1.39 ± 0.01^b^	2.02 ± 0.05^a^	0.13	0.74	0.44	0.47
γ-Terpinene	Pine, lemon	260	2.11 ± 0.12^c^	2.51 ± 0.04^b^	2.64 ± 0.06^b^	3.40 ± 0.15^a^	0.11	0.20	0.11	0.10
β-Phellandrene	Turpentine, mint	8	0.24 ± 0.01^c^	0.52 ± 0.01^b^	0.61 ± 0.01^b^	0.91 ± 0.02^a^	0.43	1.36	0.83	0.90
(+)-Limonene	Lemon, citrus, mint	10	0.56 ± 0.02^c^	0.74 ± 0.01^c^	1.46 ± 0.01^b^	1.64 ± 0.01^a^	0.80	1.54	1.57	1.30
α-Terpinene	Lemony, citrusy	85	1.81 ± 0.07^c^	2.46 ± 0.01^b^	2.54 ± 0.01^b^	3.38 ± 0.01^a^	0.30	0.61	0.32	0.32
β-Myrcene	Herbal, spice, mint	1.2	0.49 ± 0.02^c^	0.92 ± 0.02^b^	2.40 ± 0.04^a^	2.60 ± 0.06^a^	5.79	16.10	21.51	17.17
3-Carene	Pungent, herb	770	0.27 ± 0.03^d^	0.91 ± 0.02^c^	3.43 ± 0.03^a^	3.12 ± 0.07^b^	0.00	0.02	0.05	0.03
β-Thujene	Woody, spicy, citrus	980	1.81 ± 0.08^d^	2.69 ± 0.06^c^	4.20 ± 0.10^b^	5.93 ± 0.01^a^	0.03	0.06	0.05	0.05
β-Pinene	Woody, pine, minty, camphor	140	0.29 ± 0.03^c^	0.73 ± 0.02^b^	2.21 ± 0.01^a^	2.21 ± 0.02^a^	0.03	0.11	0.17	0.12
α-Pinene	Camphor, pine, earthy	14	1.26 ± 0.07^c^	1.93 ± 0.01^b^	3.37 ± 0.02^a^	3.70 ± 0.08^a^	1.27	2.89	2.59	2.09
Benzaldehyde	Bitter almond	350	0.49 ± 0.01^b^	0.38 ± 0.01^c^	1.48 ± 0.01^a^	0.33 ± 0.02^c^	0.02	0.02	0.05	0.01
1-Nonanal	Fatty, green, citrus	1	0.43 ± 0.04^a^	0.25 ± 0.00^c^	0.33 ± 0.01^b^	0.35 ± 0.02^b^	6.10	5.25	3.52	2.77
(E)-2-Heptenal	Fatty, fruity	13	0.26 ± 0.03^a^	0.09 ± 0.01^c^	0.16 ± 0.01^b^	0.17 ± 0.01^b^	0.28	0.14	0.13	0.11
1-Octanal	Green, citrus	0.7	1.02 ± 0.02^a^	0.74 ± 0.02^b^	1.15 ± 0.04^a^	0.73 ± 0.04^b^	20.67	22.11	17.67	8.24
Heptaldehyde	Oily, green, citrus	3	0.29 ± 0.01^c^	0.22 ± 0.01^c^	0.41 ± 0.01^b^	0.62 ± 0.02^a^	1.37	1.56	1.49	1.63
1-Hexanal	Green, fatty	4.5	0.30 ± 0.06^b^	0.34 ± 0.02^b^	0.39 ± 0.00^b^	0.52 ± 0.02^a^	0.95	1.60	0.94	0.91
n-Pentanal	Fermented, yogurt, pungent,	12	0.43 ± 0.06^b^	0.32 ± 0.01^c^	0.37 ± 0.00^c^	0.51 ± 0.02^a^	0.50	0.56	0.33	0.34
(E)-2-Pentenal	Green	1,500	0.30 ± 0.02^a^	0.17 ± 0.01^b^	0.25 ± 0.00^a^	0.14 ± 0.01^b^	<0.01	<0.01	<0.01	<0.01
(E)-2-Hexenal	Green, fatty	17	0.13 ± 0.01^a^	0.06 ± 0.01^b^	0.04 ± 0.01^b^	0.07 ± 0.01^b^	0.11	0.07	0.03	0.03
3-Methyl butanal	Malty, almond	1.1	0.75 ± 0.05^a^	0.70 ± 0.03^a^	0.72 ± 0.02^a^	0.42 ± 0.02^b^	9.64	13.39	7.06	3.00
Propanal	Almond, cherry, green, fruity	37	0.16 ± 0.01^b^	0.45 ± 0.03^a^	0.44 ± 0.02^a^	0.18 ± 0.01^b^	0.06	0.26	0.13	0.04
Butanal	Choking smell	9	0.04 ± 0.00^b^	0.03 ± 0.00^b^	0.09 ± 0.00^a^	0.08 ± 0.01^a^	0.07	0.07	0.11	0.07
6-Methyl-5-hepten-2-one	Citrus, green, musty	50	0.19 ± 0.01^b^	0.09 ± 0.01^c^	0.51 ± 0.02^a^	0.09 ± 0.00^c^	0.05	0.04	0.11	0.01
Isomenthone	Mint, musty, bitter	170	0.21 ± 0.02^d^	0.48 ± 0.04^b^	2.36 ± 0.03^a^	0.30 ± 0.09^c^	0.02	0.06	0.15	0.01
3-Hydroxy-2-butanone	Buttery, green, Fatty	14	1.25 ± 0.15^c^	1.60 ± 0.03^b^	1.28 ± 0.06^c^	2.34 ± 0.07^a^	1.26	2.40	0.98	1.32
2-Propanone	Pungent, irritating, floral	40,000	2.80 ± 0.04^c^	1.90 ± 0.00^d^	4.01 ± 0.11^b^	4.85 ± 0.06^a^	<0.01	<0.01	<0.01	<0.01
2-Butanone	Acetone like, fruity, camphor	35400.2	0.04 ± 0.00^c^	0.06 ± 0.01^b^	0.09 ± 0.00^a^	0.06 ± 0.01^b^	<0.01	<0.01	<0.01	<0.01
Ethyl 2-hydroxypropanoate	Sweet, fatty	50	0.30 ± 0.06^b^	0.07 ± 0.00^a^	0.04 ± 0.01^b^	0.06 ± 0.00^a^	0.09	0.03	0.01	0.01
Ethyl caprylate	Apricot, banana, pineapple	40	0.09 ± 0.00^d^	0.31 ± 0.01^b^	0.54 ± 0.02^a^	0.12 ± 0.03^c^	0.03	0.16	0.14	0.02
Bornyl acetate	Woody, pine, camphor	75	1.60 ± 0.26^b^	2.04 ± 0.12^a^	1.59 ± 0.03^b^	1.42 ± 0.11^c^	0.30	0.57	0.23	0.15
Acetic acid propyl ester	Fruity	200	–	–	0.13 ± 0.01^b^	0.76 ± 0.04^a^	–	–	0.01	0.03
Acetic acid ethyl ester	Fruity, sweet	5	–	0.11 ± 0.01^b^	0.37 ± 0.02^a^	0.31 ± 0.01^a^	–	0.46	0.79	0.48
Dimethyl trisulfide	Garlic, cooked cabbage	0.1	0.38 ± 0.03^b^	0.15 ± 0.03^c^	0.38 ± 0.03^b^	0.81 ± 0.05^a^	54.14	32.01	40.71	64.29
Dimethyl sulfide	Sulfury, oniony, sweet corn	0.3	0.26 ± 0.02^c^	0.23 ± 0.02^c^	0.31 ± 0.02^b^	0.47 ± 0.02^a^	12.06	16.06	11.06	12.47
Anethol	Anise, licorice, medicinal	15	55.92 ± 0.44^a^	52.79 ± 0.96^a^	30.51 ± 0.28^b^	24.22 ± 0.64^c^	52.71	73.69	21.89	12.78
1,8-Cineol	camphor, cool, mint	1.3	9.20 ± 0.09^c^	6.21 ± 0.21^d^	12.08 ± 0.16^b^	16.42 ± 0.25^a^	100.00	100.00	100.00	100.00
Acetic acid	Sour	22,000	4.61 ± 0.13^a^	1.73 ± 0.09^c^	1.87 ± 0.15^c^	2.96 ± 0.46^b^	<0.01	<0.01	<0.01	<0.01
1,2,4,5-Tetramethylbenzene	Rancid, sweet	NA	0.58 ± 0.00^b^	0.80 ± 0.05^b^	0.38 ± 0.04^c^	0.60 ± 0.03^b^	n.c.	n.c.	n.c.	n.c.
2-Pentylfuran	Green, fat	6	0.13 ± 0.01^d^	0.26 ± 0.01^c^	0.36 ± 0.03^b^	0.50 ± 0.02^a^	0.30	0.89	0.65	0.66

“NA” no data was reported. “–” not detected; n.c., not calculated; no odor threshold available. “a–d” different superscripts in the same row indicate significant difference (*p* < 0.05).

Among them, the ROAV values of anethol with anise, licorice, and medicinal fragrance, 3-methyl butanal with malty and almond fragrance, and 1-octanal with green and citrus fragrance in Xiaoyaozhen Hulatang samples were much higher than those in Beiwudu samples. These differences might contribute to the flavor differences between the two regions of Hulatang. Additionally, β-phellandrene, (+)-limonene, α-pinene, heptaldehyde, 1-hexanal, and 3-hydroxy-2-butanone with ROAV >1 but not as high provided pine, fruit, camphor, fatty, and green fragrance also played important modifying roles in the aroma formation of Hulatang.

### 3.6 Analysis of the characteristic aroma components based on sensory evaluation of Hulatang

**Figure 5:**
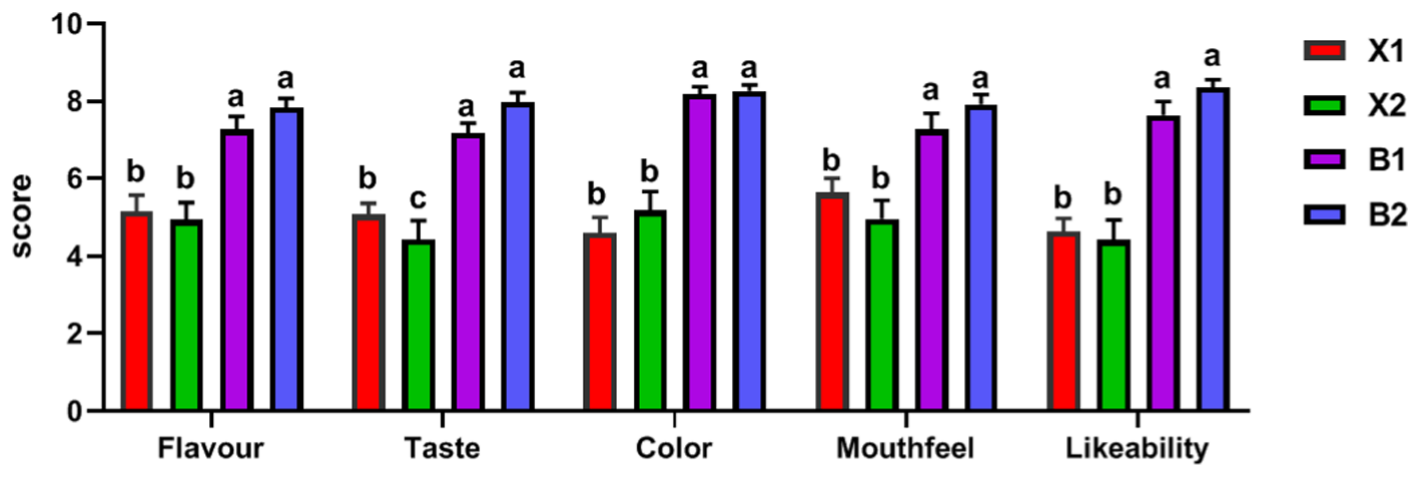
Sensory score results of Hulatang. a, b, c indicate significant differences among the four groups (*p* < 0.05).

In previous studies, a combination of instrumental analysis and sensory evaluation is necessary (43, 44). Therefore, the flavor compounds related to the senses of Hulatang were determined through sensory evaluation. As shown in Figure 5, the sensory evaluation results showed that the five sensory descriptions of the Hulatang samples were significantly different, among which the flavor, taste, color, mouthfeel, likeability scores of the samples of Beiwudu Hulatang (B1 and B2) were higher than that of the Xiaoyaozhen Hulatang samples (X1 and X2). The positive correlation between flavor, taste, color, mouthfeel, and likeability scores and the content of flavor substances with ROAV >1 was determined using the Pearson correlation coefficient. As shown in Table 2, the aroma scores in sensory evaluation were significantly positively correlated with the contents of β-phellandrene, (+)-limonene, β-myrcene, α-pinene, heptaldehyde, 1-hexanal, 3-hydroxy-2-butanone, dimethyl trisulfide, dimethyl sulfide, and 1,8-cineol (*p* < 0.05), and negatively correlated with the contents of 3-methyl butanal and anethol (*p* < 0.05). As shown in Table 1, the positively correlated substances provided camphor, pine, fruit, herbal, fatty, vegetable, and green aroma. 3-Methyl butanal provided a malty and almond flavor and played an important role in the formation of Hulatang flavor due to its low threshold (42). Anethol provided anise, licorice, and medicinal flavor, which might be derived from spices such as tangerine peel, cloves, cinnamon, anise, and fennel, and also had a low threshold (39). However, at high concentrations, anethol provides a pungent odor (45), which might be the main reason for the low sensory aroma score of Hulatang. Besides, these indicators with a significant correlation with aroma often have a similar correlation with the likeability scores, indicating that the aroma characteristics not only affect the sensory attributes of products, but also are important indicators to determine consumer preference (46, 47).

**Table 2:** Correlation analysis between the relative content of flavor substances in Hulatang and their and sensory scores.

Compounds	Flavor	Color	Mouthfeel	Taste	Likeability
Linalool	−0.076	0.017	−0.14	0.129	0.081
β-Phellandrene	0.796**	0.627*	0.569	0.572	0.674*
(+)-Limonene	0.869**	0.785**	0.677*	0.711**	0.821**
β-Myrcene	0.851**	0.769**	0.648*	0.710**	0.812**
α-Pinene	0.846**	0.745**	0.632*	0.667*	0.798**
1-Nonanal	0.145	0.158	0.209	0.116	0.124
1-Octanal	−0.062	0.123	0.027	0.176	0.112
Heptaldehyde	0.888**	0.787**	0.747**	0.660*	0.787**
1-Hexanal	0.892**	0.760**	0.783**	0.720**	0.807**
3-Methyl butanal	−0.687*	−0.503	−0.487	−0.379	−0.487
3-Hydroxy-2-butanone	0.634*	0.425	0.501	0.345	0.454
Dimethyl trisulfide	0.804**	0.709**	0.714**	0.600*	0.676*
Dimethyl sulfide	0.886**	0.744**	0.732**	0.659*	0.744**
Anethol	−0.883**	−0.805**	−0.700*	−0.722**	−0.835**
1,8-Cineol	0.878**	0.809**	0.759**	0.669*	0.797**

Asterisks indicate significant correlation coefficients. *p < 0.05, **p < 0.01.

### 3.7 Evaluation of the characteristic aroma components in Hulatang

**Figure 6:**
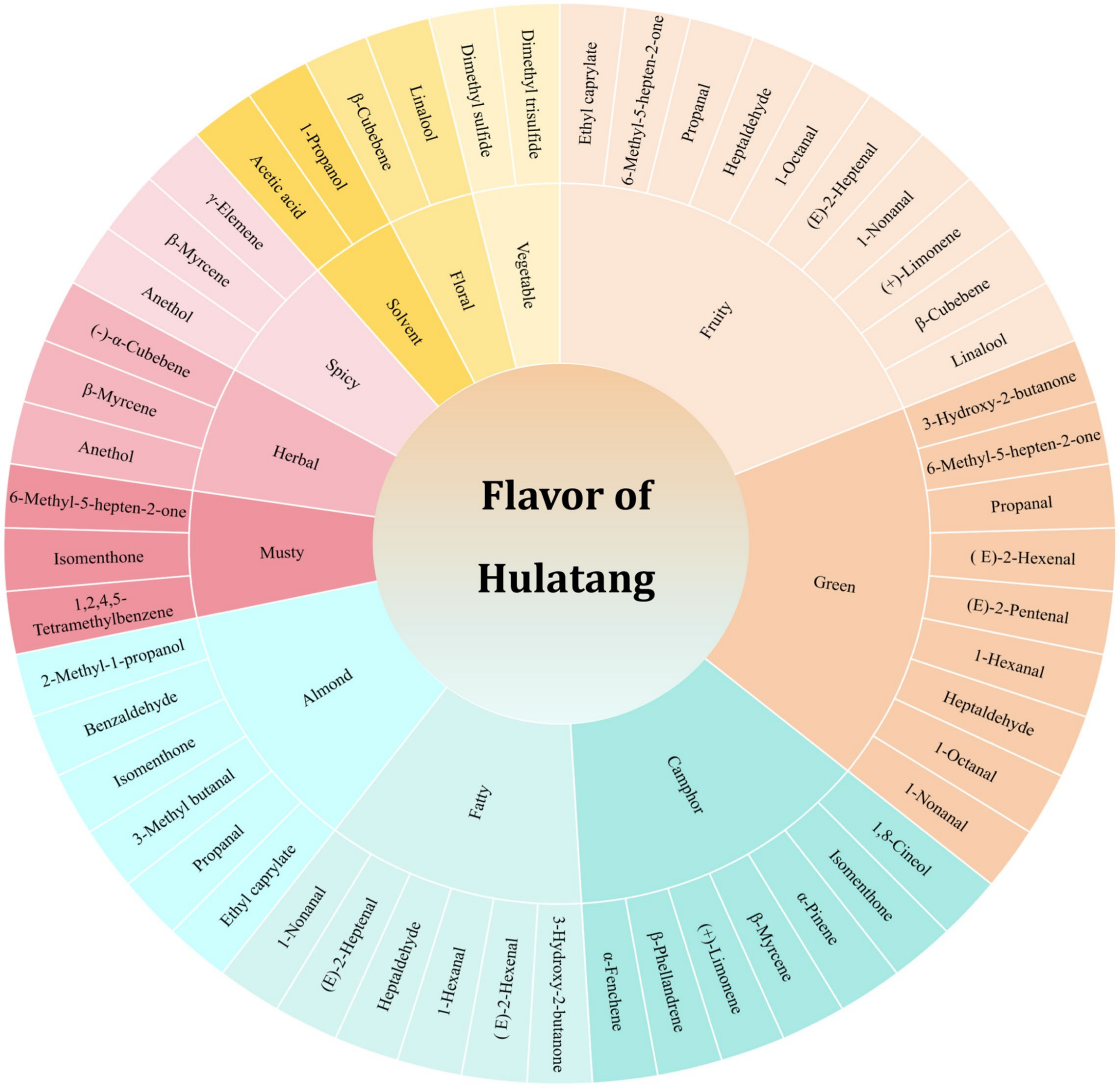
Characteristic aroma substances responsible for different aroma types.

Currently, research on the aroma profiles of Hulatang is scarce. The characteristic aroma substances selected according to different methods in the previous paper are listed in Supplementary Table S4. A total of 31 main aroma components related to Hulatang were screened, and the characteristic aroma types and their corresponding characteristic aroma substances were statistically analyzed, as shown in Figure 6. As shown in Figure 6, 7 aroma substances contributed to the characteristic camphor incense of Hulatang, including α-fenchene, β-phellandrene, (+)-limonene, β-myrcene, α-pinene, isomenthone, and 1,8-cineol, endowing Hulatang with a cool smell such as camphor and mint. The floral and fruity or fatty and green aromas of Hulatang were composed of linalool, β-cubebene, (+)-limonene, 1-nonanal, 1-hexanal, (E)-2-heptenal, 1-octanal, heptaldehyde, propanal, (E)-2-pentenal, (E)-2-hexenal, propanal, 6- methyl-5-hepten-2-one, 3-hydroxy-2-butanone and ethyl caprylate, terpenes and aldehydes accounted for the majority of the components. The two sulfide compounds of dimethyl trisulfide and dimethyl sulfide mainly contributed to the aroma of vegetables such as garlic, cooked cabbage, oniony, and sweet corn. 2-Methyl-1-propanol, benzaldehyde, isomenthone, 3-methyl butanal, propanal, ethyl caprylate, γ-elemene, β-myrcene, anethol, (−)-α-cubebene, 6-methyl-5-hepten-2-one, 1,2,4,5- tetramethylbenzene, 1-propanol, and acetic acid contributed to bitter almond, spicy, herbal, musty, solvent flavor, and other pungent odors. Overall, these different aromas contributed to the unique smell of Hulatang.

## 4 Conclusion

Aroma character is an important factor for the quality assessment of Hulatang from different regions. In this study, the volatile components and aroma characteristics of Hulatang from two major regions, Beiwudu Hulatang and Xiaoyaozhen Hulatang, were analyzed using GC-IMS and sensory evaluation methods. The GC-IMS spectrogram revealed significant differences among the two major Hulatang regions. Based on the GC-IMS results, a total of 75 compounds were detected and 49 volatile compounds were identified in Hulatang, including 5 alcohols, 15 terpenes, 12 aldehydes, 5 ketones, 5 esters, 4 ethers, and 3 other volatiles. The PCA results indicated that the differences in volatiles among the samples from different origin areas were evident. Therefore, GC-IMS combined with PCA could be a promising approach for sectarian differentiation of Hulatang. Based on the ROVAs combined with sensory analysis results, 12 key flavor substances affecting the sensory quality characteristics of Hulatang were detected. Finally, combining the VIP values obtained by OPLS-DA analysis and the ROVAs by the aroma threshold values and relative contents, 11 flavors of Hulatang were summarized, including camphor, green, almond, fatty, spicy, herbal, vegetable, fruity, floral, musty, and solvent. Nevertheless, future research must focus on determining the sensory odor characteristics of Hulatang.

## Data Availability

The original contributions presented in the study are included in the article/Supplementary material, further inquiries can be directed to the corresponding author/s.
