# Supplementary material for: Characterization of the flavor profile of Hulatang using GC-IMS coupled with sensory analysis
Source: Front Nutr. 2024 Aug 29;11:1461224. doi: 10.3389/fnut.2024.1461224 (PMC11390415; doi:10.3389/fnut.2024.1461224)
Supplement: Supplementary file 1 [file Table_1.docx]

Table S1 Information of collected samples.

| No. | Sample number | Genre | Ingredients list |
| --- | --- | --- | --- |
| 1 | X1 | Xiaoyaozhen | Corn starch, wheat flour, salt, star anise, Sichuan pepper, monosodium glutamate, white pepper, ginger, grass fruit, nutmeg, sand kernels, cinnamon, cloves, hawthorn, chili pepper, beef seasoning |
| 2 | X2 | Xiaoyaozhen | Corn starch, wheat flour, salt, monosodium glutamate, Sichuan pepper, star anise, cumin, black pepper, nutmeg, sand kernels, angelica, licorice, dried ginger, galangal, hawthorn, ginkgo seeds, cinnamon, orange peel |
| 3 | B1 | Beiwudu | Beef, gluten, corn starch, vermicelli, salt, monosodium glutamate, white pepper, black pepper, star anise, Sichuan pepper, cumin, cinnamon, nutmeg, sand kernels, cloves, galangal |
| 4 | B2 | Beiwudu | Beef, gluten, corn starch, vermicelli, salt, star anise, Sichuan pepper, pepper, cumin, cinnamon, chili, galangal, grass fruit, nutmeg, chicken powder seasoning, monosodium glutamate |

Table S2 Hulatang scoring rules.

|  | Grading criteria | | |
| --- | --- | --- | --- |
| Indicator evaluation | 7~9 | 4~6 | 1~3 |
| Flavor | the fragrance is strong, no peculiar smell, and has the unique aroma of Hulatang | no peculiar smell, but the aroma of Hulatang is slightly light | no aroma of Hulatang |
| Taste | the spicy and salty taste is moderate, and the layering is obvious | the spicy and salty taste are heavy or light, and the layering is average | the spicy and salty taste is not suitable, and there is no sense of layering |
| Color | dark brownish-yellow and evenly colored | brownish-yellow and evenly colored | light brown and uneven in color |
| Mouthfeel | delicate, soft, smooth, and non-irritating | slightly rough and irritating | rough and irritating |
| Likeability | like | neither like or dislike | dislike |

Table S3 Volatile components detected in Hulatang by GC-IMS.

| Count | Compound | CAS# | Molecule | MW | RI | Rt [sec] | Dt [a.u.] |
| --- | --- | --- | --- | --- | --- | --- | --- |
|  |  |  | formula |  |  |  |  |
|  | Alcohols |  |  |  |  |  |  |
| 1 | Linalool | C78706 | C10H18O | 154.3 | 1555.8 | 1088.264 | 1.22375 |
| 2 | 1-Propanol | C71238 | C3H8O | 60.1 | 1041 | 256.393 | 1.10884 |
| 3 | 2-Methyl-1-propanol | C78831 | C4H10O | 74.1 | 1097.5 | 305.857 | 1.17328 |
| 4 | Ethanol | C64175 | C2H6O | 46.1 | 931.1 | 189.738 | 1.1347 |
| 5 | 2-Propanol | C67630 | C3H8O | 60.1 | 901.8 | 176.488 | 1.21952 |
|  | Terpenes |  |  |  |  |  |  |
| 6 | β-Cubebene | C13744155 | C15H24 | 204.4 | 1543.8 | 1059.474 | 1.43838 |
| 7 | (-)-α-Cubebene | C17699148 | C15H24 | 204.4 | 1457.6 | 873.487 | 1.45161 |
| 8 | γ-Elemene | C29873992 | C15H24 | 204.4 | 1728.6 | 1602.99 | 1.46673 |
| 9 | α-Terpinolene | C586629 | C10H16 | 136.2 | 1276.3 | 569.787 | 1.21991 |
| 10 | α-Fenchene | C471841 | C10H16 | 136.2 | 1062.2 | 273.919 | 1.21361 |
| 11 | β-Ocimene-M | C13877913 | C10H16 | 136.2 | 1251 | 523.081 | 1.21437 |
| 12 | β-Ocimene-D | C13877913 | C10H16 | 136.2 | 1248.7 | 519.057 | 1.68739 |
| 13 | γ -Terpinene-M | C99854 | C10H16 | 136.2 | 1237.6 | 500.084 | 1.2194 |
| 14 | γ-Terpinene-D | C99854 | C10H16 | 136.2 | 1238.9 | 502.346 | 1.70721 |
| 15 | β-Phellandrene-M | C555102 | C10H16 | 136.2 | 1199.4 | 439.523 | 1.221 |
| 16 | β-Phellandrene-D | C555102 | C10H16 | 136.2 | 1198.5 | 438.167 | 1.63901 |
| 17 | (+)-Limonene-M | C138863 | C10H16 | 136.2 | 1188.7 | 423.68 | 1.22025 |
| 18 | (+)-Limonene-D | C138863 | C10H16 | 136.2 | 1190.1 | 425.71 | 1.65963 |
| 19 | α-Terpinene-M | C99865 | C10H16 | 136.2 | 1172.1 | 399.285 | 1.21883 |
| 20 | α-Terpinene-D | C99865 | C10H16 | 136.2 | 1172.9 | 400.356 | 1.72043 |
| 21 | β-Myrcene-M | C123353 | C10H16 | 136.2 | 1158.4 | 380.157 | 1.21741 |
| 22 | β-Myrcene-D | C123353 | C10H16 | 136.2 | 1158.8 | 380.765 | 1.71117 |
| 23 | 3-Carene-M | C13466789 | C10H16 | 136.2 | 1141.9 | 358.534 | 1.21741 |
| 24 | 3-Carene-D | C13466789 | C10H16 | 136.2 | 1144 | 361.173 | 1.72439 |
| 25 | β-Thujene-M | C28634891 | C10H16 | 136.2 | 1119 | 330.258 | 1.21741 |
| 26 | β-Thujene-D | C28634891 | C10H16 | 136.2 | 1115.9 | 326.6 | 1.63981 |
| 27 | β-Pinene-M | C127913 | C10H16 | 136.2 | 1101.7 | 310.576 | 1.21599 |
| 28 | β-Pinene-D | C127913 | C10H16 | 136.2 | 1102.7 | 311.619 | 1.63716 |
| 29 | α-Pinene-M | C7785708 | C10H16 | 136.2 | 1024.4 | 243.49 | 1.22037 |
| 30 | α-Pinene-D | C7785708 | C10H16 | 136.2 | 1024.6 | 243.625 | 1.66888 |
|  | Aldehydes |  |  |  |  |  |  |
| 31 | Benzaldehyde | C100527 | C7H6O | 106.1 | 1497.1 | 954.11 | 1.15552 |
| 32 | 1-Nonanal | C124196 | C9H18O | 142.2 | 1395.1 | 759.431 | 1.47249 |
| 33 | (E)-2-Heptenal | C18829555 | C7H12O | 112.2 | 1330 | 656.346 | 1.25942 |
| 34 | 1-Octanal-M | C124130 | C8H16O | 128.2 | 1289.8 | 596.303 | 1.40097 |
| 35 | 1-Octanal-D | C124130 | C8H16O | 128.2 | 1290.7 | 598.164 | 1.82789 |
| 36 | Heptaldehyde | C111717 | C7H14O | 114.2 | 1186 | 419.55 | 1.32812 |
| 37 | 1-Hexanal | C66251 | C6H12O | 100.2 | 1088.2 | 297.14 | 1.26746 |
| 38 | n-Pentanal | C110623 | C5H10O | 86.1 | 984.4 | 216.523 | 1.4188 |
| 39 | (E)-2-Pentenal | C1576870 | C5H8O | 84.1 | 1134.9 | 349.649 | 1.10984 |
| 40 | ( E)-2-Hexenal | C6728263 | C6H10O | 98.1 | 1218.6 | 468.926 | 1.18253 |
| 41 | 3-Methyl butanal | C590863 | C5H10O | 86.1 | 908.8 | 179.546 | 1.4024 |
| 42 | Propanal | C123386 | C3H6O | 58.1 | 774.9 | 128.922 | 1.14663 |
| 43 | Butanal | C123728 | C4H8O | 72.1 | 894.4 | 173.266 | 1.29081 |
|  | Ketones |  |  |  |  |  |  |
| 44 | 6-Methyl-5-hepten-2-one | C110930 | C8H14O | 126.2 | 1344.1 | 677.509 | 1.17656 |
| 45 | Isomenthone-M | C491076 | C10H18O | 154.3 | 1472.3 | 902.678 | 1.3366 |
| 46 | Isomenthone-D | C491076 | C10H18O | 154.3 | 1475.7 | 909.528 | 1.8513 |
| 47 | 2-Butanone, 3-hydroxy-M | C513860 | C4H8O2 | 88.1 | 1288.1 | 592.938 | 1.05704 |
| 48 | 2-Butanone, 3-hydroxy-D | C513860 | C4H8O2 | 88.1 | 1289.5 | 595.765 | 1.32985 |
| 49 | 2-Propanone | C67641 | C3H6O | 58.1 | 808.7 | 140.134 | 1.11483 |
| 50 | 2-Butanone | C78933 | C4H8O | 72.1 | 897.4 | 174.537 | 1.24827 |
|  | Esters |  |  |  |  |  |  |
| 51 | Ethyl 2-hydroxypropanoate | C97643 | C5H10O3 | 118.1 | 1353.4 | 691.846 | 1.13867 |
| 52 | Ethyl caprylate | C106321 | C10H20O2 | 172.3 | 1446.3 | 851.536 | 1.47412 |
| 53 | Bornyl acetate | C76493 | C12H20O2 | 196.3 | 1585.4 | 1162.798 | 1.22353 |
| 54 | Acetic acid propyl ester | C109604 | C5H10O2 | 102.1 | 977 | 212.58 | 1.47846 |
| 55 | Acetic acid ethyl ester | C141786 | C4H8O2 | 88.1 | 870.3 | 163.237 | 1.33614 |
|  | Ethers |  |  |  |  |  |  |
| 56 | Dimethyl trisulfide | C3658808 | C2H6S3 | 126.3 | 1383.7 | 740.316 | 1.29967 |
| 57 | Dimethyl sulfide | C75183 | C2H6S | 62.1 | 722.7 | 113.265 | 0.96181 |
| 58 | Anethol-M | C104461 | C10H12O | 148.2 | 1652.9 | 1352.812 | 1.22442 |
| 59 | Anethol-D | C104461 | C10H12O | 148.2 | 1651.4 | 1348.179 | 1.75754 |
| 60 | 1,8-Cineol-M | C470826 | C10H18O | 154.3 | 1199.3 | 439.398 | 1.29543 |
| 61 | 1,8-Cineol-D | C470826 | C10H18O | 154.3 | 1207.3 | 451.332 | 1.72247 |
|  | Others |  |  |  |  |  |  |
| 62 | Acetic acid-M | C64197 | C2H4O2 | 60.1 | 1460.3 | 878.697 | 1.05369 |
| 63 | Acetic acid-D | C64197 | C2H4O2 | 60.1 | 1459 | 876.238 | 1.15001 |
| 64 | 1,2,4,5-Tetramethylbenzene | C95932 | C10H14 | 134.2 | 1456.1 | 870.595 | 1.22161 |
| 65 | 2-Pentylfuran | C3777693 | C9H14O | 138.2 | 1229 | 485.636 | 1.25125 |
|  | Unidentified |  |  |  |  |  |  |
| 66 | 1 | unidentified | * | 0 | 1548.3 | 1070.204 | 1.93609 |
| 67 | 2 | unidentified | * | 0 | 1656.4 | 1363.271 | 1.3876 |
| 68 | 3 | unidentified | * | 0 | 1416.7 | 797.098 | 1.23575 |
| 69 | 4 | unidentified | * | 0 | 1219.7 | 470.654 | 1.67946 |
| 70 | 5 | unidentified | * | 0 | 1041.8 | 257.095 | 0.94875 |
| 71 | 6 | unidentified | * | 0 | 1149.7 | 368.552 | 1.58362 |
| 72 | 7 | unidentified | * | 0 | 1124.3 | 336.593 | 1.08872 |
| 73 | 8 | unidentified | * | 0 | 1246.3 | 514.893 | 1.12394 |
| 74 | 9 | unidentified | * | 0 | 917.1 | 183.283 | 1.2765 |
| 75 | 10 | unidentified | * | 0 | 1457 | 872.181 | 1.9218 |

Note: “CAS#” an alias of CAS number which indicates the unique numerical identification number of a substance. “MW” the molecule weight of the volatiles. “RI” retention Index. “Rt” retention time. “Dt” drift time in the drift tube.

Table S4 The key aroma substances in Hulatang screened by different methods.

| Screening method | Key aroma substances |
| --- | --- |
| VIP>1 | 1-Octanal, 6-Methyl-5-hepten-2-one, (E)-2-Heptenal, 1,2,4,5-Tetramethylbenzene, (E)-2-Pentenal, Benzaldehyde, 1-Nonanal, Isomenthone, (-)-α-Cubebene, Linalool, Propanal, Ethyl caprylate, γ-Elemene, Acetic acid, 3-Hydroxy-2-butanone, 2-Methyl-1-propanol, 1-Propanol, β-Cubebene, α-Fenchene, (E)-2-Hexenal |
| ROAV>1 | Linalool, β-Phellandrene, (+)-Limonene, β-Myrcene, α-Pinene, 1-Nonanal, 1-Octanal, Heptaldehyde, 1-Hexanal, 3-Methyl butanal, 3-Hydroxy-2-butanone, Dimethyl trisulfide, Dimethyl sulfide, Anethol, 1,8-Cineol |
| Significantly correlated with odor sensory | β-Phellandrene, (+)-Limonene, β-Myrcene, α-Pinene, Heptaldehyde, 1-Hexanal, 3-Methyl butanal, 3-Hydroxy-2-butanone, Dimethyl trisulfide, Dimethyl sulfide, Anethol, 1,8-Cineol |
